# Hyaluronan synthase 2 expressed by cancer-associated fibroblasts promotes oral cancer invasion

**DOI:** 10.1186/s13046-016-0458-0

**Published:** 2016-11-25

**Authors:** Ziwen Zhang, Detao Tao, Ping Zhang, Xue Liu, Yuchao Zhang, Jie Cheng, Hua Yuan, Laikui Liu, Hongbing Jiang

**Affiliations:** 1 Jiangsu Key Laboratory of Oral Diseases, Nanjing Medical University, 136 Hanzhong Road, Nanjing, 210029 Jiangsu Province China; 2 Department of Oral and Maxillofacial Surgery, Affiliated Hospital of Stomatology, Nanjing Medical University, 136 Hanzhong Road, Nanjing, 210029 Jiangsu Province China; 3 Department of Basic Science of Stomatology, Affiliated Hospital of Stomatology, Nanjing Medical University, 136 Hanzhong Road, Nanjing, 210029 Jiangsu Province China

**Keywords:** Cancer-associated fibroblasts, Hyaluronan synthase 2, Oral squamous cell carcinoma, Tumor microenviroment, Invasion, Metastasis

## Abstract

**Background:**

Hyaluronan synthases (HAS) control the biosynthesis of hyaluronan (HA) and critically modulate the tumor microenviroment. Cancer-associated fibroblasts (CAFs) affect the progression of a tumor by remolding the matrix. However, little is known about the role of HAS from CAFs in this process. This study aimed to determine the role of hyaluronan synthase 2 (HAS2) from CAFs in the progression of oral squamous cell carcinoma (OSCC) invasion.

**Methods:**

HAS isoforms 1, 2, and 3 in paired sets of CAFs and normal fibroblasts (NFs) were examined by real-time PCR, and the expression of HAS2 and α-SMA in OSCC tissue sections was further evaluated using immunohistochemical staining. Furthermore, we used a conditioned culture medium model to evaluate the effects of HAS2 from CAFs on the invasion and epithelial-mesenchymal transition (EMT) of the oral cancer cells Cal27. Finally, we compared the expression of matrix metalloproteinases (MMPs) and tissue inhibitors of metalloproteinases (TIMPs) between CAFs and NF, and between CAFs with or without HAS2 knockdown using an antibody array and western blotting.

**Results:**

CAFs expressed higher levels of HAS2 than the paired NFs. HAS2 expression was consistent with α-SMA-positive myofibroblasts in the stroma of OSCC, and these were significantly correlated advanced clinical stages and cervical lymph node metastasis. Knocking down HAS2 with a specific siRNA or treatment with a HAS inhibitor markedly attenuated CAF-induced invasion and EMT of Cal27 cells. Higher MMP1 and lower TIMP1 levels were detected in the supernatants of CAFs relative to NFs. Knocking down HAS2 could decrease the expression of MMP1 and increase that of TIMP1 in CAFs.

**Conclusions:**

HAS2 is one of the key regulators responsible for CAF-mediated OSCC progression and acts by modulating the balance of MMP1 and TIMP1.

**Electronic supplementary material:**

The online version of this article (doi:10.1186/s13046-016-0458-0) contains supplementary material, which is available to authorized users.

## Background

Oral squamous cell carcinoma (OSCC) is prone to metastasis through lymphatic channels, which leads to cancer-related mortality and an unfavorable prognosis [1]. During this intricate process, the malignant epithelial cells interact with the adjacent stromal cells and the extracellular matrix (ECM) to support their migration and invasion. Cancer-associated fibroblasts (CAFs), which are also known as myofibroblasts and are characterized by α-smooth muscle actin (α-SMA) expression, are the stromal cells involved in producing a tumor cell niche in coordination with other cell types such as macrophages to fuel cancer overgrowth and spreading [2]. Accumulating evidence has revealed that CAFs can be used as a reliable marker to predict tumor progression and metastasis in various human cancers [3–8]. Our preliminary study has suggested roles for CAFs in cancer metastasis and the survival of patients with OSCC and suggested that the α-SMA-positive myofibroblasts in OSCC facilitate the epithelial-mesenchymal transition (EMT) and formation of the pro-metastatic microenvironment [9]. However, the underlying mechanisms that are responsible for CAF-mediated OSCC dissemination remain unknown.

In contrast to normal fibroblasts (NFs), CAFs are able to secrete a broad spectrum of bioactive molecules in a paracrine manner. These molecules promote the growth and invasion of tumor cells [10–13]. Matrix metalloproteinases (MMPs) are bioactive enzymes that are differentially produced by these two types of fibroblasts [11, 14]. It has been established that MMPs degrade certain substrates that regulate cell-cell adhesion and cell-matrix adhesion and that this process is crucial for tumor cell migration and EMT [15]. Recent studies have revealed that MMPs and the natural endogenous tissue inhibitors of metalloproteinases (TIMPs) orchestrate the multiple diverse steps of tumor progression [16, 17]. Furthermore, the subtle balance between MMPs and TIMPs in the tumor microenvironment plays vital roles in tumor invasion and metastasis.

Hyaluronan (HA) is a glycosaminoglycan component of the extracellular matrix that mediates microenvironmental signals [18]. Three members of Hyaluronan synthase family (HAS1, HAS2 and HAS3) dictate the biosynthesis of HA and are critical for embryonic development, tumorigenesis and inflammation [19]. These enzymes have been reported to affect the patient outcome in numerous cancers by modulating the activities of MMPs and TIMPs [20, 21]. For example, up-regulation of HAS2 in breast cancer promotes the invasive potential of tumor cells by suppressing TIMP1 [22]. Inhibition of HAS2 and/or HAS3 decreased the invasion by colon carcinoma cells, probably by inducing both the expression and activity of MMP-7 [23]. These studies emphasize that HAS-mediated regulation of MMPs and TIMPs is a critical factor that affects the function of these proteins in cancerous cells. However, these processes are largely undefined in the tumor stromal cells. Thus, it was of great interest to determine whether HAS isozymes from CAFs influence the invasion and EMT of the OSCC by affecting the balance of MMPs and TIMPs in the tumor microenvironment.

In this study, we investigated whether HAS from CAFs influenced the invasion and EMT of the OSCC and determined the potential molecular mechanisms that underlie this process. We first confirmed that the HAS2 expression is higher in CAFs by comparing the levels of HAS1-3 in the paired CAFs and NFs from three different OSCC patients. We then analyzed the relationship between HAS2 expression in the tumor stroma and clinical/pathological characteristics of the OSCC. Furthermore, we investigated the effects of HAS2 on CAF-induced migration, invasion and EMT of OSCC using an in vitro conditioned culture medium system. Our findings indicated that HAS2 is one of the key regulators responsible for CAF-mediated OSCC progression and that it acts by modulating the balance between MMP1 and TIMP1.

## Methods

### Patients and specimens

Between January 2011 and October 2013, a total of 48 patients with primary OSCC underwent radical resections at our hospital. Sixteen of the patients provided paired adjacent non-cancer oral mucosa from the negative surgical margins. The primary sites of the OSCC were the tongue (*n* = 20), buccal mucosa (*n* = 11), gingiva (*n* = 12) and oral floor (*n* = 5). All of the surgical specimens were reviewed and analyzed after the study protocol was approved by the ethics committee of Nanjing Medical University.

### Immunohistochemistry

The paraffin-embedded specimens were sectioned at 5 μm thickness, and the sections were baked at 37 °C overnight. After deparaffinization using xylol and rehydration with a graded series of ethanol solutions, the sections were treated with 3% H_2_O_2_ plus 1% triton in paraformaldehyde for 10 min at room temperature followed by incubation with normal goat serum (Boster, Wuhan, China) for another 30 min. Next, the sections were incubated with primary antibodies for α-SMA and HAS2 (Abcam, Cambridge, MA, USA) at 4 °C overnight. After being washed in phosphate buffered saline (PBS), a Polink2-plus reagent kit (Zhongsan Jinqiao Biotechnology, Beijing, China) was used to detect the bound antibodies. Finally, the slides were stained with 2% 3,3-diaminobenzidine (DAB, Zhongsan Jinqiao Biotechnology) in 50 mM Tris-buffer (pH 7.6) containing 0.3% hydrogen peroxide. The slides were counterstained with Meyer’s hematoxylin. The negative controls were prepared by replacing the primary antibody with PBS. The immunostaining results were evaluated independently by two researchers.

### Evaluation of immunoreactivity

HAS2 and α-SMA immunostaining were initially evaluated under low-power magnification to identify several areas with relatively high staining. Subsequently, high-power magnification was used to evaluate the tumor stromal cells in each section. The expression of HAS2 and α-SMA was graded according to the percentage of positive cells: low (<25%) and high (≥25%). The final results were determined by the highest grade of each slice.

### Cell culture

Fresh tissues were obtained from 3 patients with OSCC (one each from the tongue, gingiva and buccal mucosa) who underwent radical surgical resection at our hospital. The fresh cancer tissues were used to obtain primary CAFs and the corresponding normal mucosa was excised at the negative margin to obtain paired primary NFs. Briefly, the fresh tissues were sliced to 1 mm^3^ sizes, dispersed into a culture flask, and allowed to adhere for 3 h. The tissue blocks were then maintained in DMEM supplemented with 10% fetal bovine serum (FBS) until the cells grew into confluent monolayers. After 2–3 passages, the purified fibroblasts were retained and used for this study before their 5th passage. The oral cancer cell line Cal27 was obtained from American Type Culture Collection (ATCC) and cultured in DMEM/F12 medium with 10% FBS at 37 °C in an atmosphere containing 5% CO2.

### Quantitative real-time PCR

The total RNA of the cells was extracted using TRIzol reagent (Invitrogen, San Diego, CA, USA). The reverse transcription to cDNA was carried out using the PrimeScript RT kit (TaKaRa Holdings, Inc., Kyoto, Japan). The primers, Taqman probes and SYBR Premix Ex Taq™ were from TaKaRa. The amplification of the target genes was performed according to the manufacturer’s protocols. The relative expression of each target gene was normalized to the level in the control samples using the comparative 2^-ΔΔCT^ method. The primer sequences (forward and reverse) are listed in Additional file 1: Table S1.

### Western blotting

The harvested cells were lysed on ice for 30 min in RIPA Lysis Buffer (Beyotime, Shanghai, China) containing 100 mM PMSF (Beyotime) and centrifuged at 12000 × g for 5 min. The protein lysate supernatants were mixed with loading buffer, separated by SDS-PAGE and transferred to polyvinyldifluoride membranes (Millipore Corporation, Billerica, MA, USA). The final amount of protein loaded in each well was 20 μg. The membranes were blocked with 5% non-fat milk at room temperature, incubated with primary antibodies at 4 °C overnight, washed in PBST and incubated with the secondary peroxidase-conjugated antibodies. Finally, the protein bands on the membranes were visualized using chemiluminescence reagents (WBKLS0100; Millipore Corporation, Billerica, MA, USA). The supplier and the specifications of each primary antibody were as follows: rabbit anti-HAS2 monoclonal antibody and goat anti-TIMP-1 polyclonal antibody (Santa Cruz Biotechnology, Santa Cruz, CA, USA; 200 μg/ml); mouse anti-β-actin polyclonal antibody (Bioworld; Louis Park, MN, USA; 1 mg/ml); rabbit anti-E-cadherin polyclonal antibody (Bioworld; 1 mg/ml); rabbit anti-vimentin monoclonal antibody (CST) and mouse anti-MMP1 polyclonal antibody (Boster).

### Immunofluorescence

The cells were pre-seeded onto sterile cover slips and grown to 60% confluence, then fixed with 4% paraformaldehyde for 20 min, permeabilized with 0.2% Triton X-100 (Sigma) for 15 min, and then incubated with normal goat serum and the primary antibodies at 4 °C overnight. The cells were then washed with PBS, incubated with the secondary antibody conjugated with TRITC (Bioworld; Louis Park, MN, USA) (1:100) for 1 h and subsequently incubated with DAPI (Beyotime, 1:1000) for 5 min. The primary antibodies used were vimentin (Cell Signaling, 1:200), cytokeratin (Bioworld, 1:200) and HAS2 (Abcam, 1:200).

### Knockdown of HAS2 expression by small interference RNA

An HAS2-targeted small interference RNA (siRNA) (Sigma, St. Louis, MO, USA) was used to obtain specific suppression of HAS2 in CAFs. CAFs were permitted to grow to 60% confluence and were infected with HAS2-siRNA or a scrambled siRNA using Lipofectamine 2000 (Invitrogen) for 6 h according to the manufacturer’s instructions. The medium was then changed to complete culture medium, and CAFs were permitted to grow for another 48 h before being used for the experiments. The sequences targeted by siRNA were as follows: HAS2-siRNA-1, sense: 5’CCAGUAUCAGUUUGGUUUAdTdT3’, antisense: 5’UAAACCAAACUGAUACUGGdTdT3’; HAS2-siRNA-2, sense: 5’GGAUUAAAGUUGUCAUGGUdTdT3’, antisense: 5’ACCAUGACAACUUUAAUCCdTdT3’; Scramble siRNA, sense: 5’UUCUCCGAACGUGUCACGUTT3’, antisense: 5’ACGUGACACGUUCGGAGAATT3’.

### Collection of conditioned medium

When they achieved confluency, the corresponding cells were re-suspended and counted, and 4 × 10^6^ cells were added to a 25 cm^2^ flask with 6 ml of FBS-free medium. After incubation for 48 h, the conditioned medium was collected and used immediately. In the case of the collection of the CM from CAFs that were treated with 4-MU (Sigma), which was dissolved and stored in DMSO, the 4-MU was removed after 48 h of treatment to avoid direct effects on the tumor cells.

### Wound-healing and invasion assay

Cal27 cells were seeded in a 6-well plate and cultured in growth medium until they reached 90% confluency. A sterile pipette was used to produce a linear scratch on the cell monolayer. After wounding, the non-adherent cells and debris were carefully removed, and the cells were incubated with either serum-free medium or CM from either NFs or CAFs for 36 h. The migrated cells were visualized in a specific region. The invasion assay was performed using matrigel-coated transwell inserts. A total of 5 × 10^4^ Cal27 cells in 250 μl of serum-free medium or in the CM from NFs or CAFs were seeded into the upper chamber, and 750 μl of medium containing 10% FBS was added to the lower chamber to stimulate chemotaxis. After incubation for 36 h at 37 °C in 5% CO2, the chambers were fixed in 4% paraformaldehyde for 15 min, stained with 0.05% crystal violet for 15 min and the numbers of cells in five random fields at 100× magnification were counted using a microscope.

### Bromodeoxyuridine (BrdU) assay

The cells were incubated for 24 h with 0.03 mg/ml BrdU (Sigma), fixed with 4% paraformaldehyde containing 2 M HCl, blocked for 1 h with goat serum containing 0.5% Triton at 37 °C, and then incubated with BrdU antibodies (1:300, Proteintech, USA) at 4 °C overnight. Next, the cells were washed with PBS, incubated for 40 min with a secondary antibody labeled with Cy3 (Bioworld, 1:100) and stained with DAPI. The BrdU-positive cells in six randomly selected fields were counted using a microscope at 200 ×.

### RayBio antibody array

The medium supernatants were collected from NFs, CAFs, scrambled siRNA-treated CAFs and HAS2 siRNA-treated CAFs. All of these samples were evaluated by RayBiotech Testing Services using the human MMP-related antibody array (RayBio, Norcross, GA, USA). Briefly, 1 ml of a 1:5 dilution of the sample was loaded onto each cytokine membrane. The samples were incubated overnight at 4 °C to allow the binding of the target cytokines to the capture antibodies, followed by several wash steps. A secondary biotin-labeled detection antibody was added to bind to the captured antigens. The cytokine-antibody-biotin complex was visualized by the addition of FITC-conjugated avidin. The intensity of the signals was quantified using the Axon GenePix laser scanner system. QAH-MMP-1 software was used to analyze the cytokine expression intensity. This program automatically compares the fluorescence intensity of the samples to standard curves to determine the concentrations of MMPs and TIMPs. Only values within the valid interval were subjected to statistical analysis. The valid intervals for all of the targets are presented in Additional file 2: Table S2.

### Statistical analysis

All data were analyzed using SPSS 17.0 software. Chi-Square test or Fisher’s exact test was used to analyze the data from the four-fold table in this article. Student’s *t*-test was used to analyze the results from the RayBio Antibody Array. One-way ANOVA was used to analyze the data from the three groups described above, and the SNK q-test was used for additional multiple comparisons if necessary. A value of *P* < 0.05 was considered to be statistically significant.

## Results

### HAS2 expression is higher in CAFs than in NFs

**Fig. 1:**
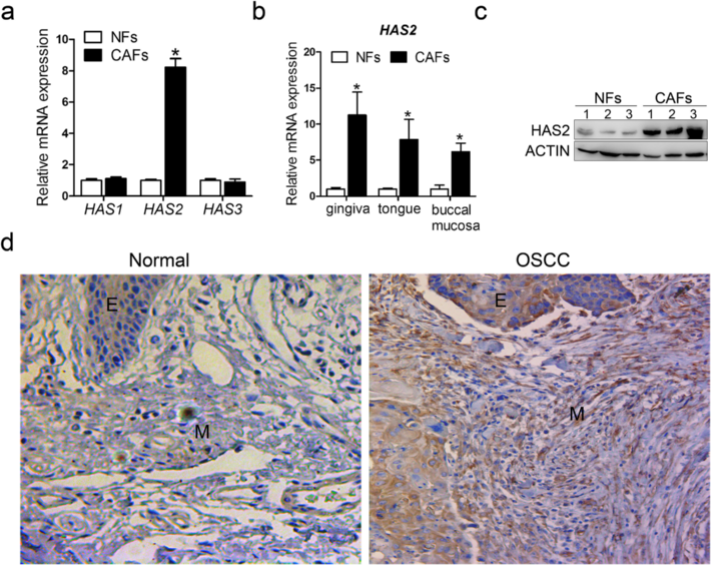
Higher expression of HAS2 in CAFs than in matched NFs. **a** The relative expression of HAS1, 2, 3 was measured by real-time PCR using SYBR *Green* in three matched cell samples and displayed as the average of the 2^-ΔΔCT^ values. **b** HAS2 expression was assayed by real-time PCR using SYBR *Green* in three individual matched cell samples. **c** HAS2 expression was assayed by western blotting in three matched cell samples. **d** HAS2 expression (*brown color*) in the OSCC and normal oral mucosa was examined by immunohistochemistry staining (400x magnification). *p < 0.05 in (**a**) and (**b**). **e**: epithelia; M: stroma

To determine the abundance of HAS isoforms in CAFs from OSCCs and the paired NFs from the same patients, fresh OSCC samples were acquired from three patients by macroscopic dissection using the following morphological mapping: CAFs from the actual tumors were isolated from the epicenter of the tumor tissue, and the matched NFs were isolated from distal normal tissue located at least 10 mm from the tumor margins. Three pairs of matched CAFs and NFs were successfully isolated and cultured in vitro as indicated by the fibroblast characteristics including spindle-shaped morphology, strong expression of the fibroblastic marker vimentin and the absence of expression of the epithelial marker cytokeratin in each type of cells (Additional file 3: Figure S1a). Meanwhile, strong expression of α-SMA, a key marker for the identification of CAFs, was observed in CAFs but was absent in NFs (Additional file 3: Figure S1b, c). Among the three members of the HAS family, the abundance of HAS2 were significantly enriched in CAFs. Importantly, this isoform was also overexpressed in CAFs compared to the matched NFs, whereas there were no differences between CAFs and NFs in the expression of HAS1 or HAS3 (Fig. 1a, b).

We next focused on HAS2 for further investigations. In accordance with the PCR results, CAFs expressed a markedly higher level of the HAS2 protein than NFs (Fig. 1c). Consistent with this result, stronger HAS2 staining was visualized in the stromal fibroblasts from the OSCC samples than in the normal tissue (Fig. 1d). Although both the OSCC and the normal epithelium showed HAS2 immunoreactivity, significant differences in the HAS2 expression were only observed in the stroma (*P* < 0.05) (Table 1). Together, these results suggested that CAFs expressed higher levels of HAS2 and that HAS2 from the tumor stromal microenvironment may be associated with tumorigenesis.

**Table 1:** Differential expression of HAS2 in OSCC and normal oral mucosa

	Epithelia		*P* value	Stroma		*P* value
	cancer	normal		cancer	normal	
HAS2(High)	45	15	1.000	25	2	**0.008**
HAS2(Low)	3	1		23	14	

Bold values signify *P* value < 0.05. Fisher’s exact test

### HAS2 expression is associated with OSCC progression

**Fig. 2:**
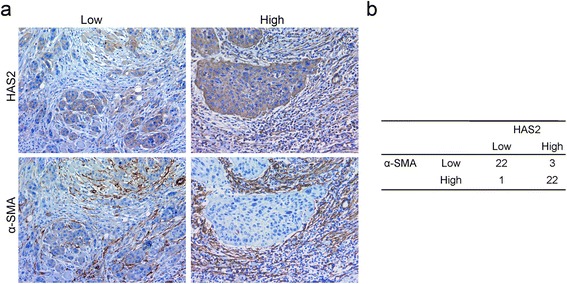
Positive association of the expression of HAS2 and α-SMA in OSCC. **a** Within serial sections of the same tissue, the expression of HAS2 and α-SMA (*brown color*) was showed by immunohistochemistry staining (400x magnification). **b** Qualitative staining scores of HAS2 and α-SMA in a total of 48 tumor samples

Although there is no specific marker for CAFs, α-SMA has been widely used to characterize these cells [24]. To further examine HAS2 expression in the α-SMA-positive CAFs, we performed immunohistochemical staining on serial sections. As shown in Fig. 2a, HAS2 expression was positively correlated with that of α-SMA in the tumor stroma, which implied that CAFs might be the main source of HAS2 in the tumor stroma of the OSCC (Fig. 2b). Notably, the abundance of stromal HAS2 was found to be significantly correlated with advanced clinical stages and cervical lymph node metastases (Table 2). These findings indicated that the higher expression of HAS2 in CAFs was associated with progression and metastases of the OSCC.

**Table 2:** Relation between HAS2, α-SMA expression levels and clinicopathological variables

Variable	HAS2			α-SMA		
	High	Low	*P* value	High	Low	*P* value
HD			1.000			0.777
I	15	13		14	14	
II + III	10	10		9	11	
TNM status			**0.021**			**0.000**
I + II	8	15		5	18	
III + IV	17	8		18	7	
PLN status			**0.020**			**0.038**
Negative	11	18		10	19	
Positive	14	5		13	6	

α-SMA, α-smooth muscle actin; *HD* histological differentiation, *TNM* tumor-node-metastases, *PLN* pathologic lymph node. Bold values signify *P* value < 0.05. Chi-Square test

### CAFs promotes migration, invasion and EMT of OSCC cells

**Fig. 3:**
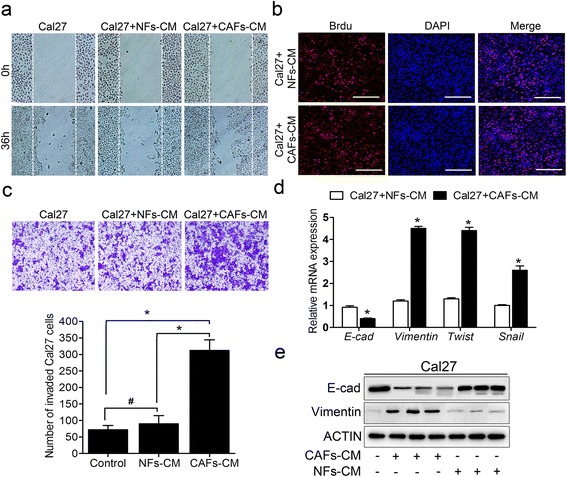
CAFs promoted migration, invasion and EMT of Cal27 cells. **a** Representative images of CAF-CM promoting Cal27 cell migration compared to control and NFs-CM for 36 h incubation. **b** Representative images of Cal27 cell proliferation by BrdU incorporation assay after treated with CAFs-CM or NFs-CM. **c** The cells invading the filters coated with the Matrigel after 36 h (*upper panel*); and the quantification of the cell invasion in five randomly chosen fields (*bottom panel*). **d** The expression of EMT-associated genes *E-cad*, *Vimentin*, *Twist*, and *Snail* was measured by real-time PCR using SYBR *Green*. **e** The expression of E-cad and Vimentin was measured using western blotting. *p < 0.05; ^#^ *p* > 0.05 in (**c**) and (**d**). CM: conditioned medium

Previous investigations have confirmed that CAFs fuel the invasiveness and spreading of OSCC cells by direct crosstalk or a paracrine process [3, 25, 26]. To evaluate the role of paracrine effects, we treated Cal27 cells with CAF- or NF-conditioned medium (CM). As expected, the migration and invasion of the Cal27 cells were remarkably enhanced after being cultured in CAF-CM compared with NF-CM (Fig. 3a, c). We performed a proliferation assay to exclude bystander effects due to increased cell proliferation, which further verified that CAF-CM enhanced the migration and invasion of the Cal27 cells (Fig. 3b). CAF-CM also induced EMT in the Cal27 cells, which was characterized by a remarkable decrease in the level of E-cadherin and increases in vimentin, Twist and Snail. However, the matched NF-CM failed to induce similar effects (Fig. 3d, e). Collectively, these results suggest that CAFs from the tumor microenvironment might promote the invasion and metastasis cascades of the cancer cells by triggering the EMT process in a paracrine manner.

### Knocking down HAS2 decreases CAF-induced migration, invasion and EMT of the OSCC cells

**Fig. 4:**
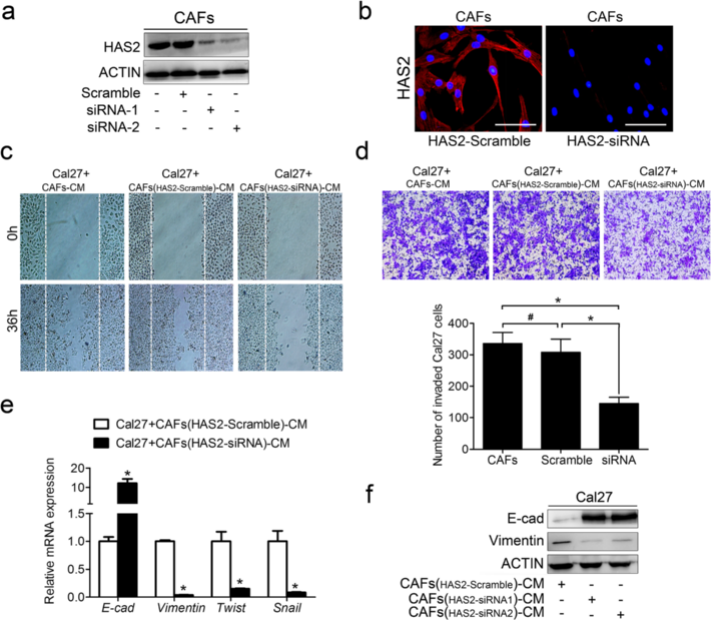
Knocking down HAS2 in CAFs reduced the migration, invasion and EMT of the Cal27 cells. The expression of HAS2 was evaluated in CAFs that had been transfected with a scrambled siRNA or an HAS2-targeted siRNA by western blotting (**a**) and immunofluorescence (**b**). Representative images of the migration (**c**) and invasion (**d**) of the Cal27 cells that were cultured with CM from CAFs, CAFs with a scrambled siRNA or CAFs with HAS2-targeted siRNA for 36 h. **e** The expression of EMT-associated genes was measured by real-time PCR using SYBR *Green*. **f** The expression of E-cad and Vimentin was analyzed using western blotting. *p < 0.05; ^#^ *p* > 0.05 in (**d**) and (**e**). Bars in (**b**): 100 μm. siRNA: short interfering RNA; CM: conditioned medium

To assess whether HAS2 participated in CAF-induced migration, invasion and EMT of cancer cells, CAFs were transfected with HAS2-specific siRNA or a scrambled siRNA. As expected, the efficiency of knockdown of HAS2 was above 75% for two HAS2-targeted siRNAs (#1 and #2) (Additional file 4: Figure S2a). HAS2 knockdown neither impacted the expression of HAS1 and HAS3, nor the proliferation and apoptosis of CAFs (Additional file 4: Figure S2a–c). The knockdown of HAS2 in CAFs was confirmed by western blotting and immunofluorescence (Fig. 4a, b). Compared with the scrambled siRNA and the control, HAS2 depletion robustly attenuated CAF-induced migration and invasion of the Cal27 cells (Fig. 4c, d), which suggested that knocking down HAS2 ameliorated CAF-derived paracrine stimulation of the Cal27 cell behaviors. Similarly, knocking down HAS2 in CAFs prevented the EMT-associated phenotypic changes in the Cal27 cells that were induced by CAFs (Fig. 4e, f). These findings suggest that HAS2 might be a key mediator that is involved in the process of CAF-promoted OSCC progression.

### An HAS inhibitor suppresses CAF-induced migration, invasion and EMT of OSCC cells

**Fig. 5:**
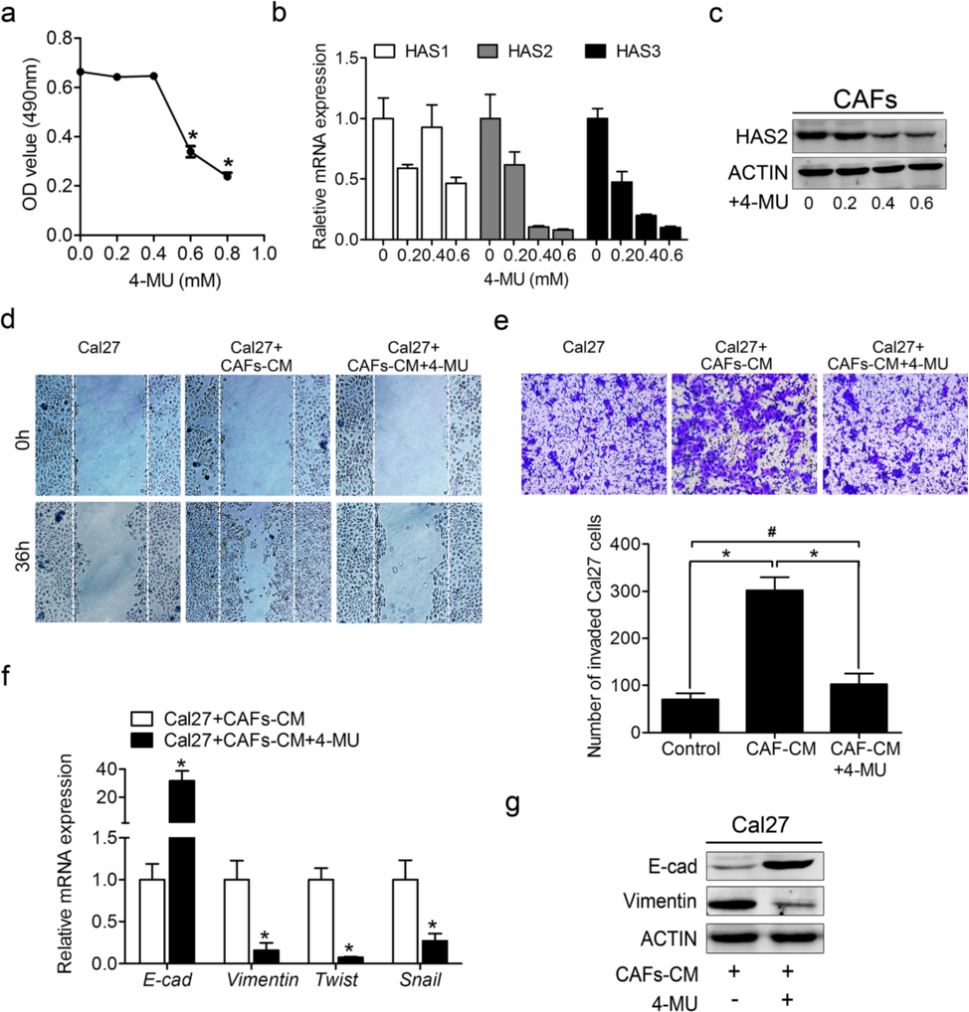
Treatment of CAFs with 4-MU reduced the migration, invasion and EMT of the Cal27 cells. **a** The viability of CAFs after 48 h of treatment with various concentration of 4-MU assessed as the OD following the MTT assay. **b** The levels of the HAS isoforms in CAFs after 48 h of treatment with various concentration of 4-MU were measured by real-time PCR using SYBR *Green*. and shown as the average 2^-ΔΔCT^ values. **c** The expression of HAS2 in CAFs cultured with various concentrations of 4-MU was determined by western blotting. **d** Representative images of the migration (**d**) and invasion (**e**) of Cal27 cells cultured with serum-free medium, CM from CAFs, or CM from CAFs that had been treated with 0.4 mM 4-MU. **f** The expression of EMT-associated genes was measured by real-time PCR using SYBR *Green*. **g** The expression of E-cad and Vimentin was examined by western blotting. *p < 0.05; ^#^ *p* > 0.05 in (**a**), (**e**) and (**f**). 4-MU: 4-Methylumbelliferone; CM: conditioned medium

To further explore the roles of CAF-derived HAS2 in cancer cell migration and invasion, the HA synthesis inhibitor 4-methylumbelliferone (4-MU) was used to elucidate the underlying mechanism. First, we determined the concentration of 4-MU appropriate for the exposure of CAFs. Our results showed that the cell viability did not change significantly when 4-MU was added at concentrations less than 0.4 mM but declined at higher concentrations (Fig. 5a). The expression of HAS2 was potently inhibited when more than 0.4 mM 4-MU was added (Fig. 5b, c). Thus, we chose 0.4 mM as an appropriate concentration in the following experiments. As shown in Fig. 5d and e, the migration and invasion of the Cal27 cells was enhanced when they were cultured with CAF-CM. However, this effect was greatly attenuated when 4-MU was added. Furthermore, the increases in the EMT markers of the Cal27 cells were partially abrogated following 4-MU exposure (Fig. 5f, g).

### HAS2 regulates CAF-promoted OSCC progression by altering the balance of MMP1 and TIMP1

**Fig. 6:**
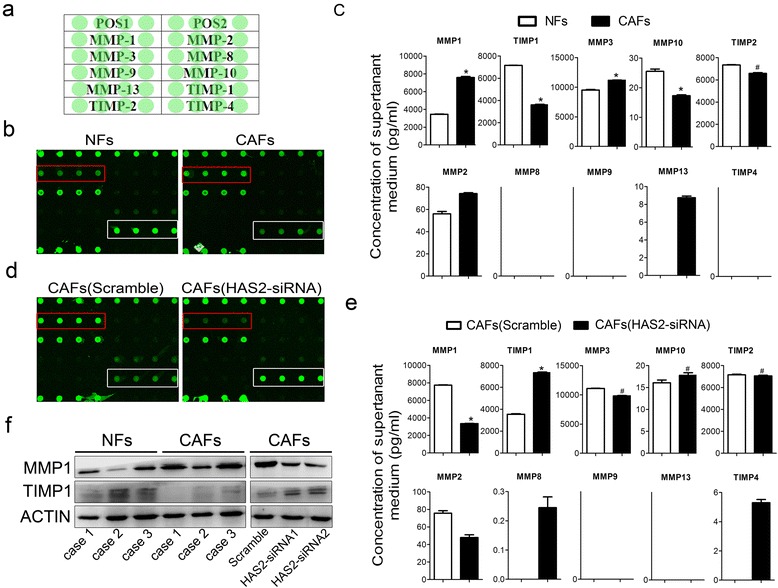
The expression of MMP1 and TIMP1 is regulated by HAS2. **a** General scheme of the application of the samples; POS1 and 2 represent the positive controls. **b** Fluorescent images of the antibody assays for NFs and CAFs. **c** The fluorescence intensity was automatically compared to standard curves and was translated into the absolute concentration values in b. **d** Fluorescent images of the antibody assays for CAFs with scramble and CAFs with HAS2-siRNA. **e** The fluorescence intensity was translated into the absolute concentration values in d. **f** The expression of MMP1 and TIMP1 was analyzed using western blotting. *p < 0.05; ^#^ *p* > 0.05 in (**c**) and (**e**)

Mounting evidence has demonstrated that MMP family members are critically involved in tumor invasion and EMT and that they are valuable factors for the diagnosis and prognosis of OSCC patients. Totals of seven members of the MMP family and three members of the TIMP family were measured using the RayBio Human MMP-Related Antibody Array (Fig. 6a). We first evaluated the supernatant media from NFs and CAFs and found that compared to NFs, CAFs expressed higher levels of MMP1 and MMP3 and lower levels of MMP10 and TIMP1 (Fig. 6b, c). To elucidate the role of HAS2 in the regulation of MMPs and TIMPs, we examined the supernatant of the media from CAFs with/without HAS2 knockdown. The results showed that knocking down HAS2 remarkably decreased the MMP1 level and increased the TIMP1 level in the media from CAFs, but the changes in MMP3 and MMP10 were not significant (Fig. 6d, e). To further confirm the potential roles of MMP1 and TIMP1 in CAFs, three paired sets of CAFs and NFs were examined by western blotting, and the level of MMP1 was higher in CAFs than in the matched NFs whereas TIMP1 showed the opposite pattern (Fig. 6f). As expected, knocking down HAS2 remarkably decreased the expression of MMP1 and increased the expression of TIMP1 in CAFs (Fig. 6f). Collectively, these results indicated that HAS2 led to disturbances in the levels of MMP1 and TIMP1 which may, at least in part, be responsible for CAF-promoted OSCC progression.

## Discussion

In the current study, we provided novel evidence that CAFs expressed higher levels of HAS2 than the paired NFs. We also revealed that HAS2, which is expressed by CAFs, plays a potential role in promoting the invasion of oral cancer cells. Importantly, we clarified the pivotal role of HAS2, which probably led to disruption in the expression of MMP1 and TIMP1 and consequently promoted cancer progression. Our findings extend the current understanding of the molecular mechanisms underlying the tumor-promoting functions of CAFs.

CAFs are the primary stromal cell type. These cells promote cancer progression through the secretion of growth factors, matrix-degrading enzymes, and pro-angiogenic factors in various solid cancer types [27–30]. In this study, we further characterized CAFs from OSCC in vitro and in vivo and provided the first evidence for a significant role for HAS2 from CAFs in promoting the EMT, invasion and metastases of OSCC. Two distinct CAF subtypes have been shown to promote the invasion of tumor cells by different mechanisms including a high production of hyaluronan or the synthesis of TGF-β1 [27]. Although the role of CAF-derived TGF-β1 in promoting tumor progression has been well studied, the role of CAF-derived hyaluronan in this progression remains largely unexplored [31]. Our data clearly showed that the HAS2 level is associated with CAF-mediated promotion of the migration, invasion and EMT of the OSCC cells. These results suggested a pivotal role of CAF-derived hyaluronan in promoting tumor progression. However, more studies are needed to investigate the function of the specific hyaluronan that is synthesized by HAS2.

The overexpression of HAS2 has been observed in some solid cancers in which it is associated with more aggressive tumor phenotypes [30–34]. However, it is still largely unknown whether there is a correlation between the HAS2 expression levels in CAFs and tumor aggressiveness. Although there is no specific marker for CAFs, α-SMA has been widely used to characterize these cells [9]. In our study, we found that HAS2 expression was consistently associated with the α-SMA-positive myofibroblasts in the stroma of OSCC and both of these were significantly correlated with advanced clinic stages and cervical lymph node metastasis. These findings extend the previous knowledge that CAF-derived HAS2 promotes oral cancer progression.

Cancers have sometimes been described as resembling chronically unhealed wounds, and myofibroblasts may be essential for the invasive growth and associated with poor prognosis [35]. Our data revealed that CAFs expressed a higher level of HAS2 than NFs and that HAS2 was positively correlated with α-SMA. Indeed, HA generation is essential for persistence of myofibroblast phenotype in lung fibroblasts, and HA deprivation by inhibition of HAS2 reduces the expression of α-SMA [18]. Furthermore, over-expression of HAS2 in oral fibroblasts modifies their response to TGF-β1 [36]. These data imply that CAFs in oral cancer may secrete a larger amount of HA by over-expressing HAS2 to maintain their phenotype. Future studies should address whether cytokines or growth factors such as TGF-β1, PDGF and IL-1β in the tumor environment [37–40] contribute to activating quiescent fibroblasts.

Our findings confirm that the HAS2-mediated MMP1 and TIMP1 changes are most likely responsible for the mechanisms underlying CAF-mediated promotion of the migration, invasion and EMT of oral cancer cells. The mechanism for CAF-induced EMT of cancer cells has been well explained by evidence that the TIMP family members control the ECM integrity and cell surface protein landscape through post-translational inhibition of MMPs [41]. In our study, we found that Cal27 cells undergo the EMT process when cultured in CAF-CM, and the EMT phenotype could be blocked when HAS2 of CAFs was knocked down or when the cells were treated with an HAS inhibitor. Of note, HAS2-mediated downregulation of TIMP1 may be responsible for the phenotypic changes of the CAFs. TIMP loss in fibroblasts elicits the myofibroblast phenotype that is associated with the conversion of stromal fibroblasts into CAFs [42]. TIMP-knockout fibroblasts release metalloproteinase activity within the tumor-stromal compartment. These experiments showed that complete TIMP loss is sufficient for the acquisition of CAFs functions [38]. In accordance with these findings, we found that TIMP1 expression was lower in CAFs than in the matched NFs and that knocking down HAS2 rescued TIMP1 expression and CAF function. Further studies are needed to elucidate how HAS2 mediates TIMP1 expression.

## Conclusions

In summary, the data presented here demonstrate that HAS2, which is expressed by CAFs, may facilitate the migration, invasion and EMT of oral cancer cells by disturbing the balance between MMP1 and TIMP1. Thus, CAF-derived HAS2 may be a new therapeutic target against oral cancer.

## Additional files

Additional file 1: Table S1. Sequence information for the specific primers used. (DOCX 13 kb)
Additional file 2: Table S2. The valid interval of each index. (DOCX 12 kb)
Additional file 3: Figure S1. Characteristics of CAFs and NFs isolated from OSCC. (DOCX 1083 kb)
Additional file 4: Figure S2. Effects of CM from CAFs and CAFs of siHAS2 on cal27 cell viability and apoptosis. (DOC 7125 kb)

## Abbreviations

4-MU: 4-Methylumbelliferone
CAFs: Cancer-associated fibroblasts
EMT: Epithelial-mesenchymal transition
HAS: Hyaluronan synthase
MMPs: Matrix metalloproteinases
NFs: Normal fibroblasts
OSCC: Oral squamous cell carcinoma
siRNA: Small interfering RNA
TIMPs: Tissue inhibitors of metalloproteinases
α-SMA: α-smooth muscle actin
