# Supplementary material for: Hyaluronan synthase 2 expressed by cancer-associated fibroblasts promotes oral cancer invasion
Source: J Exp Clin Cancer Res. 2016 Nov 25;35:181. doi: 10.1186/s13046-016-0458-0 (PMC5123319; doi:10.1186/s13046-016-0458-0)
Supplement: Additional file 1: Table S1. — Sequence information for the specific primers used. (DOCX 13 kb) [file 13046_2016_458_MOESM1_ESM.docx]

Table S1 Sequence information for the specific primers used

| **Gene name** | **Forward primer** | **Reverse primer** |
| --- | --- | --- |
| HAS1 | 5’GGTGGGGACGTGCGGATC3’ | 5’ATGCAGGATACACAGTGGAAGTAG3’ |
| HAS2 | 5’TGAACAAAACAGTTGCCCTTT3’ | 5’TTCCCATCTATGACCATGACAA3’ |
| HAS3 | 5’CTCTACTCCCTCCTCTATATGTC3’ | 5’AACTGCCACCCAGATGGA3’ |
| E-cadherin | 5’CAGTCAAAAGGCCTCTACGG3’ | 5’GTGTATGTGGCAATGCGTTC-3’ |
| Vimentin | 5’TGCCCTTAAAGGAACCAATGA3’ | 5’AAGGTGACGAGCCATTTCCTC3’ |
| Snail | 5’CCCAGTGCCTCGACCACTAT3’ | 5’GCTGGAAGGTAAACTCTGGATTAGA3’ |
| Twist | 5'GGAGTCCGCAGTCTTACGAG3’ | 5'TCTGGAGGACCTGGTAGAGG3’ |
| GAPDH | 5’GGAGTCAACGGATTTGGT3’ | 5’GTGATGGGATTTCCATTGAT3’ |
