# Supplementary material for: Hyaluronan synthase 2 expressed by cancer-associated fibroblasts promotes oral cancer invasion
Source: J Exp Clin Cancer Res. 2016 Nov 25;35:181. doi: 10.1186/s13046-016-0458-0 (PMC5123319; doi:10.1186/s13046-016-0458-0)
Supplement: Additional file 2: Table S2. — The valid interval of each index. (DOCX 12 kb) [file 13046_2016_458_MOESM2_ESM.docx]

Table S2 The valid interval of each index.

| (pg/ml) | MMP-1 | MMP-2 | MMP-3 | MMP-8 | MMP-9 | MMP-10 | MMP-13 | TIMP-1 | TIMP-2 | TIMP-4 |
| --- | --- | --- | --- | --- | --- | --- | --- | --- | --- | --- |
| LOD | 253.7 | 111.6 | 214.4 | 13.0 | 25.0 | 4.5 | 27.7 | 32.4 | 46.9 | 42.6 |
| Max | 200,000 | 100,000 | 60,000 | 40,000 | 60,000 | 10,000 | 40,000 | 40,000 | 40,000 | 100,000 |

LOD: the limit of detection Max: the highest standards
