# Supplementary material for: Hyaluronan synthase 2 expressed by cancer-associated fibroblasts promotes oral cancer invasion
Source: J Exp Clin Cancer Res. 2016 Nov 25;35:181. doi: 10.1186/s13046-016-0458-0 (PMC5123319; doi:10.1186/s13046-016-0458-0)
Supplement: Additional file 3: Figure S1. — Characteristics of CAFs and NFs isolated from OSCC. (DOCX 1083 kb) [file 13046_2016_458_MOESM3_ESM.docx]

**Fig. S1**

**
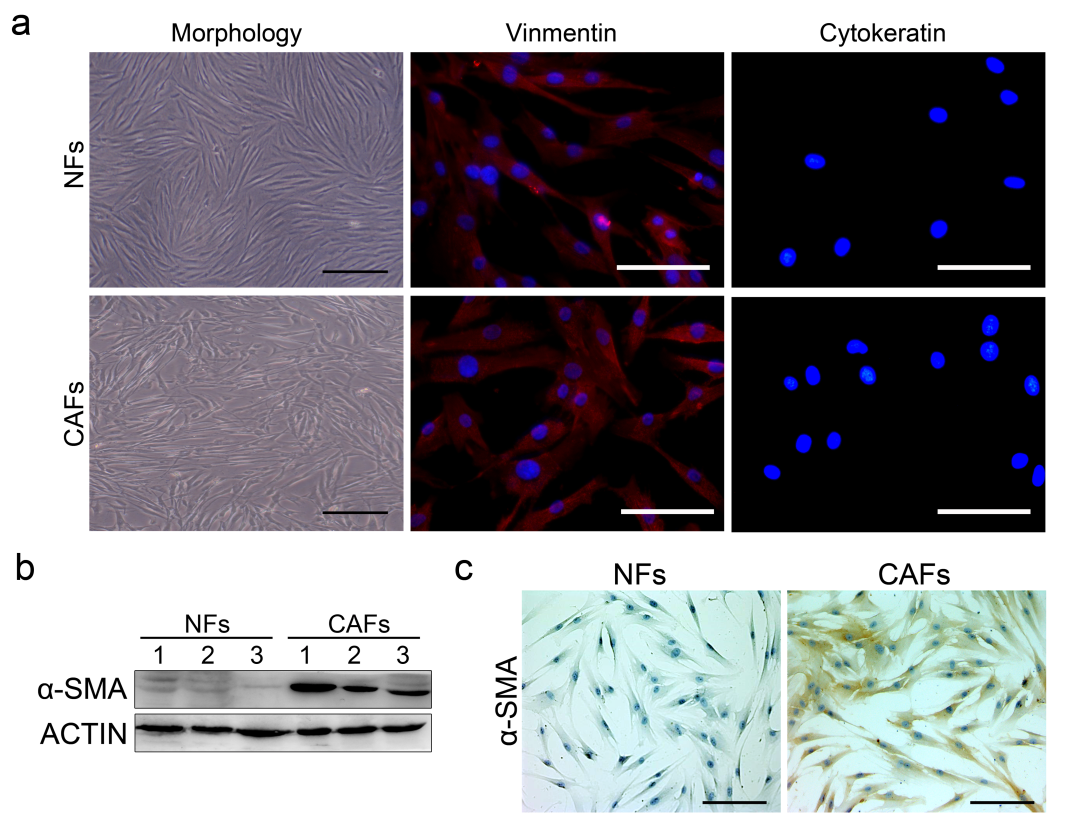
**

**Fig.S1 Characteristics of CAFs and NFs isolated from OSCC**

Fibroblasts isolated from OSCC tissues exhibited spindle-like appearance under phase-contrast microscopy. Both CAFs and NFs showed positive staining for mesenchymal marker vimentin and negative staining for the epithelial marker cytokeratin. (Nuclei, blue; vimentin, red) (Fig.S1a). Expression of α-SMA in CAFs and NFs were detected by western blot (Fig.S1b) and immunocytochemical staining (Fig.S1c).
