# Supplementary material for: Hyaluronan synthase 2 expressed by cancer-associated fibroblasts promotes oral cancer invasion
Source: J Exp Clin Cancer Res. 2016 Nov 25;35:181. doi: 10.1186/s13046-016-0458-0 (PMC5123319; doi:10.1186/s13046-016-0458-0)
Supplement: Additional file 4: Figure S2. — Effects of CM from CAFs and CAFs of siHAS2 on cal27 cell viability and apoptosis. (DOC 7125 kb) [file 13046_2016_458_MOESM4_ESM.doc]

Fig. S2

Fig. S2 Effects of CM from CAFs and CAFs of siHAS2 on cal27 cell viability and apoptosis
Knockdown of HAS2 neither impacted on expression of HAS1 and HAS3 nor proliferation and apoptosis of CAFs. The mRNA levels of three HAS isforms were measured by Real time PCR (Fig.S2a). The results indicated that HAS2 were down-regulated. Neither HAS1 nor HAS3 were impacted after transfection with HAS2 siRNA. There was no significant difference in the cell proliferation between CAFs group and HAS2 siRNA group according to the MTT assay results (Fig.S2b). The apoptosis rate of CAFs and CAFs with siHAS2 was 2.91% versus 3.04% (Fig.S2c). These data suggest knockdown HAS2 do not influence the viability and apoptosis of CAFs.


Materials and methods
Cell proliferation and apoptosis
Cell proliferation was assessed by MTT assay. Briefly, 1×10^3^/well cells were seeded into 96-well plates. After 0, 1, 3, 5 or 7 days of culture, 5 mg/ml MTT (Sigma) was added to each well to incubate for 4 h at 37 °C. Then the medium was removed and the crystalline was resolved by DMSO. The value of optical density at 490 nm was recorded in an automatic enzyme-linked immunosorbent assay reader (ELx 800; BioTek Instruments Inc., Winooski, VT, USA). Cell apoptosis was assessed using Annexin Vcombined PI methods and analysed by Flow cytometry at fundermental laboratary of Nanjing medical university.
